# Dengue Infection Complicated by Hemophagocytic Lymphohistiocytosis: Experiences From 180 Patients With Severe Dengue

**DOI:** 10.1093/cid/ciz499

**Published:** 2019-06-12

**Authors:** Foong Kee Kan, Cheng Cheng Tan, Tatiana Von Bahr Greenwood, Khairil E Khalid, Premaa Supramaniam, Ida Hed Myrberg, Lian Huat Tan, Jan-Inge Henter

**Affiliations:** 1 Department of Medicine, Hospital Sultanah Aminah, Johor Bahru, Malaysia; 2 Department of Anesthesiology and Intensive Care, Hospital Sultanah Aminah, Johor Bahru, Malaysia; 3 Childhood Cancer Research Unit, Department of Women’s and Children’s Health, Karolinska Institute; 4 Theme of Children’s and Women’s Health, Karolinska University Hospital, Stockholm, Sweden; 5 Clinical Research Centre, Hospital Sultanah Aminah, Johor Bahru; 6 Sunway Medical Centre, Petaling Jaya, Selangor, Malaysia

**Keywords:** dengue infection, hemophagocytic lymphohistiocytosis, corticosteroids, etoposide, multiorgan failure

## Abstract

**Background:**

Globally, ~500 000 people with severe dengue (SD) require hospitalization yearly; ~12 500 (2.5%) die. Secondary hemophagocytic lymphohistiocytosis (sHLH) is a potentially fatal hyperinflammatory condition for which HLH-directed therapy (as etoposide and dexamethasone) can be life-saving. Prompted by the high mortality in SD and the increasing awareness that patients with SD may develop sHLH, our objectives were to (1) determine the frequency of dengue-HLH in SD, (2) describe clinical features of dengue-HLH, (3) assess mortality rate in SD and dengue-HLH, and (4) identify mortality-associated risk factors in SD.

**Methods:**

A 5-year retrospective single-center study in all adult patients with SD admitted to a tertiary intensive care unit in Malaysia.

**Results:**

Thirty-nine of 180 (22%) patients with SD died. Twenty-one of 180 (12%) had HLH defined as an HLH probability ≥70% according to histo score (HScore); 9 (43%) died. Similarly, 12 of 31 (39%) fulfilling ≥4 and 7 of 9 (78%) fulfilling ≥5 HLH-2004 diagnostic criteria died. Peak values of aspartate aminotransferase (AST), alanine aminotransferase, lactate dehydrogenase, and creatinine correlated to fatality (odds ratios [ORs], 2.9, 3.4, 5.8, and 31.9; all *P* < .0001), as did peak ferritin (OR, 2.5; *P* = .0028), nadir platelets (OR, 1.9; *P* = .00068), hepatomegaly (OR, 2.9; *P* = .012), and increasing age (OR, 1.2; *P* = .0043). Multivariable logistic regression revealed peak AST (OR, 2.8; *P* = .0019), peak creatinine (OR, 7.3; *P* = .0065), and SOFA (Sequential Organ Failure Assessment) score (OR, 1.4; *P* = .0051) as independent risk factors of death.

**Conclusions:**

Be observant of dengue-HLH due to its high mortality. A prospective study is suggested on prompt HLH-directed therapy in SD patients with hyperinflammation and evolving multiorgan failure at risk of developing dengue-HLH.

## Teaser

Patient with severe dengue (SD) may develop secondary hemophagocytic lymphohistiocytosis (HLH), which is potentially fatal but treatable. We report an SD mortality of 22% and dengue-HLH mortality ~40%. Consider prompt HLH-directed therapy in patients with, or at risk of, dengue-HLH.

The global incidence of dengue infection has grown dramatically and has been estimated at 390 million infections yearly [1]. This mosquito-borne virus infection occasionally develops into a potentially lethal state called severe dengue (SD) [2]. Yearly, ~500 000 individuals with SD require hospitalization (~2.5% die), according to the World Health Organization (WHO) [2].

Hemophagocytic lymphohistiocytosis (HLH) is a hyperinflammatory condition that comes in primary (genetic) and secondary (acquired) forms [3]. The most common causes of secondary HLH (sHLH) are infections, neoplasms, and autoimmune diseases; the most common infection-associated HLH is caused by Epstein-Barr virus (EBV-HLH) [4]. Primary HLH has a near 100% fatality without adequate treatment. However, in international studies (HLH-94/HLH-2004) survival increased from ~0% to 60% with HLH-directed treatment including dexamethasone and the cytotoxic drug etoposide [5, 6]. Survival in severe EBV-HLH has also improved markedly with similar HLH-directed therapy [7–10].

Despite prompt and appropriate fluid management, dengue mortality is considerable and some patients with SD develop sHLH (dengue virus–associated HLH [dengue-HLH]) [11, 12]. Thus, dengue-HLH is a subset of SD, and of sHLH. Importantly, sHLH is potentially fatal but treatable if appropriate therapy is initiated in a timely manner [13–19]. Criteria for SD include severe liver and central nervous system (CNS) involvement [20], findings that together with high persistent high fever, cytopenia, hyperferritinemia, and hemophagocytosis may be indicative of HLH, often defined by the HLH-2004 diagnostic criteria and sometimes by the diagnostic HScore [21, 22].

In Malaysia, 385 758 dengue infections were reported during 2000–2010 [23]. The Ministry of Health Malaysia reported 83 849 dengue infections in 2017, of whom 177 (0.21%) patients died. The Malaysian Registry of Intensive Care showed that dengue infection was among the top 5 diagnoses bringing patients to the intensive care unit (ICU) during 2013–2017. Prompted by the high mortality and that SD may develop into sHLH, we performed a 5-year retrospective single-center study in all adult patients with SD admitted to a tertiary care ICU with the objectives to (1) determine the frequency of dengue-HLH, (2) describe clinical features of dengue-HLH, (3) assess mortality rate, and (4) identify mortality-associated risk factors in SD. Interestingly, a recent meta-analysis on 122 dengue-HLH patients reported a fatality rate of 15% and highlighted the need to explore potential relationships between specific dengue findings and dengue‐associated HLH [19].

## METHODS

All adults (≥18 years) with SD and positive dengue NS1 antigen, and/or immunoglobulin (Ig) M (IgM) enzyme-linked immunosorbent assay (ELISA) and/or IgG ELISA [24] admitted to the multidisciplinary ICU at Hospital Sultanah Aminah, Johor Bahru, during 2010–2014 were included in the study, which was approved by the Medical Ethics and Research Committee, Malaysia. Severe dengue was defined according to the WHO 2009 classification, as follows:

1. severe plasma leakage leading to (a) shock or (b) fluid accumulation and respiratory distress;
2. severe bleeding (as evaluated by the treating physician); and/or
3. severe organ involvement of (a) liver (aspartate aminotransferase [AST] and/or alanine aminotransferase [ALT] ≥1000 U/L), (b) CNS (impaired consciousness), and/or (c) heart and other organs [20]. Impaired consciousness was in this study defined by confusion, seizures, drowsiness, aggressiveness, and/or altered behavior (excluding delirium and other non–dengue-related reasons).

Hemophagocytic lymphohistiocytosis was retrospectively evaluated and defined by 2 different approaches: (1) fulfilling ≥4 of the 8 HLH-2004 diagnostic criteria (without genetics, natural killer (NK)–cell activity, and soluble interleukin-2 receptor) and (2) HScore with 70% or greater HLH-probability [21, 22]. The following laboratory parameters were studied: hemoglobin, hematocrit, leukocytes, neutrophils, platelets, AST, ALT, lactate dehydrogenase (LDH), ferritin, triglycerides, fibrinogen, and creatinine. Clinical parameters included fever, splenomegaly, and hepatomegaly, and hemophagocytosis if evaluated. In addition, Acute Physiology, Age, Chronic Health Evaluation (APACHE) II and Simplified Acute Physiology Score (SAPS) II, ICU severity-of-disease scoring systems to predict mortality risk, as well as Sequential Organ Failure Assessment (SOFA) score, an organ-failure scoring system, were evaluated [25, 26]. Data were retrieved from the ICU registry, case report forms, and medical files.

### Statistical Analysis

Statistical analysis was performed using IBM SPSS Statistics version 25 and R version 3.5.0. Descriptive statistics include frequencies, proportions, medians, and ranges. The distribution of continuous variables (AST, ALT, LDH, ferritin, triglycerides, fibrinogen, platelet count, creatinine, age on admission, APACHE II score, SAPS II score, and SOFA score) was assessed by histograms and normal qq-plots. Continuous variables were log-transformed using the natural logarithm, except for age and APACHE II, SAPS II, and SOFA scores. Fisher’s exact test was used to assess association between mortality and different cutoff levels of AST and ferritin, respectively.

Univariable logistic regression was used to evaluate risk factors for death. An odds ratio (OR) of 2.0 for a binary variable (ie, sex) indicates that the odds of dying are doubled and for a continuous variable that the odds are doubled for a 1-unit change in the variable (if log-transformed, a 1-unit change in its natural logarithm). The linearity assumption (log-transformed) of continuous predictors was checked by fitting models with restricted cubic splines using 3 knots (function rcs in R-package rms). A clear deviation from linearity was found for fibrinogen, which was excluded from multivariable regression due to many missing values (80%).

The parameters with the highest significance in univariable logistic regression were candidates for multivariable regression. For parameter pairs with high correlation (Pearson’s correlation coefficient >0.6), only 1 parameter per pair was chosen to avoid multicollinearity. Candidates chosen for multivariable logistic regression were severe bleed, severe leak, severe organ involvement, age, SOFA score, AST, platelet count, and creatinine. Ferritin and HLH status were not included due to many missing values. The multivariable logistic regression model was fitted using backward regression, with Akaike’s information criterion as selection criteria. Univariable logistic regression was used to evaluate having an HScore probability of HLH of 70% or greater and having 4 or more HLH-2004 criteria, respectively. Missing data were excluded listwise from the logistic regression models. In the multivariable logistic regression models, only complete cases were included. Sensitivity and specificity of the selected multivariable model were evaluated using a receiver operating characteristic (ROC) curve and assessing the area under the curve (AUC). An optimal cutoff was calculated as the value maximizing the sum of sensitivity and specificity.

Two-sample *t* tests were used when comparing log-transformed levels of AST, ALT, and ferritin between patients with and without corticosteroid treatment.

## RESULTS

### Frequency and Clinical Characteristics of Severe Dengue

Of 8802 ICU admissions during the 5-year period, 287 had dengue infections but 9 had missing records, leaving 278 patients for analysis. Of these, 197 (71%) had SD. After excluding 17 patients aged less than 18 years, 180 patients were analyzed (103 men; 57%) (Table 1). Most were NS1 positive (n = 137); of the rest, 37 were IgM and IgG positive, 1 IgG positive only, and 5 IgM positive only. A total of 108 patients had severe plasma leakage, 64 severe bleeding, and 99 severe organ involvement (Supplementary Table S1). Among the latter, 68 had severe liver involvement, 35 CNS involvement, and 38 involvement of heart and other organs (some had multiple organ involvement) (Supplementary Table S2). Altogether, 87 of 180 (48%) had severe involvement of the liver and/or CNS, a clinical picture resembling HLH.

**Table 1.** Presentation of All 180 Patients With Severe Dengue and Separated Into Survivors and Nonsurvivors

	All (N = 180)	Survivors (n = 141)	Nonsurvivors (n = 39)
Female	77 (42.8)/103 (57.2)^a^	56 (39.7)/85 (60.3)	21 (53.8)/18 (46.2)
Splenomegaly	7 (4.6)/144 (95.4); 29	4 (3.5)/109 (96.5); 28	3 (7.9)/35 (92.1); 1
Hepatomegaly	33 (21.9)/118 (78.1); 29	19 (16.8)/94 (83.2); 28	14 (36.8)/24 (63.2); 1
Hemophagocytosis	16 (80.0)/4 (20.0); 160	7 (70.0)/3 (30.0); 131	9 (90.0)/1 (10.0); 29
Leak	108 (60.0)/72 (40.0)	79 (56.0)/62 (44.0)	29 (74.4)/10 (25.6)
Bleed	64 (35.6)/116 (64.4)	41 (29.1)/100 (70.9)	23 (59.0)/16 (41.0)
Severe organ involvement	99 (56.3)/77 (43.8); 4	62 (45.3)/75 (54.7); 4	37 (94.9)/2 (5.1)
Severe organ involvement (liver)	68 (38.9)/107 (61.1); 5	36 (26.5)/100 (73.5); 5	32 (82.1)/7 (17.9)
Severe organ involvement (CNS)	35 (19.4)/145 (80.6)	25 (17.7)/116 (82.3)	10 (25.6)/29 (74.4)
Severe organ involvement (heart and other)	38 (21.1)/142 (78.9)	15 (10.6)/126 (89.4)	23 (59.0)/16 (41.0)
Intubation and ventilation	70 (38.9)/110 (61.1)	31 (22.0)/110 (78.0)	39 (100.0)/0 (0.0)
Inotropic support	55 (30.6)/125 (69.4)	16 (11.3)/125 (88.7)	39 (100.0)/0 (0.0)
Continuous veno-venous hemodiafiltration	32 (17.8)/148 (82.2)	4 (2.8)/137 (97.2)	28 (71.8)/11 (28.2)
Corticosteroid treatment	25 (13.9)/155 (86.1)	12 (8.5)/129 (91.5)	13 (33.3)/26 (66.7)
Age at hospital admission, y	34.9 (18.2–84.3)^b^	34.4 (18.2–79.7)	43.6 (19.3–84.3)
Length of hospitalization, d	5.9 (0.2–60.1)	6.1 (2.7–60.1)	2.6 (0.2–43.7)
Length of ICU, d	2.6 (0.1–60.0)	2.7 (0.2–60.0)	2.1 (0.1–43.3)
Lowest platelets, ×10^9^/L	15.5 (0–154)	17 (0–154)	9 (1–99)
Peak triglycerides, mmol/L	2.33 (0.7–11.2); 117	2.49 (0.87–11.2); 96	2.065 (0.7–9.7); 21
Lowest fibrinogen, g/L	2.15 (1–5.7); 144	2.35 (1.3–3.8); 117	1.55 (1–5.7); 27
Peak ferritin, μg/L	22 236 (565 to >100 000); 104	20 356 (565 to >100 000); 86	72 000 (2420 to >100 000); 18
Peak aspartate aminotransferase, U/L	510 (22–35 427); 4	356 (22–9861); 4	4799 (113–35 427)
Peak alanine aminotransferase, U/L	246 (12–8330); 1	181.5 (12–3344); 1	1892 (81–8330)
Peak lactate dehydrogenase, U/L	1203 (249–21 591); 7	843.5 (249–9982); 7	6244 (492–21 591)
Peak creatinine, μmol/L	100 (20–1440)	90 (20–650)	300 (80–1440)
APACHE II score	12 (1–46); 2	10 (1–26)	28 (7–46); 2
SAPS II score	20 (6–106); 2	17 (6–59)	62 (9–106); 2
SOFA score	6 (0–20); 2	5 (0–14)	15 (4–20); 2

Abbreviations: APACHE, Acute Physiology, Age, Chronic Health Evaluation; CNS, central nervous system; ICU, intensive care unit; SAPS, Simplified Acute Physiology Score; SOFA, Sequential Organ Failure Assessment.

^a^No. (%) yes/no; no. missing (if applicable).

^b^Median (range); no. missing (if applicable).

The median peak AST, ALT, and LDH levels for the 180 patients were 510 U/L (range, 22–35 427 U/L), 246 U/L (12–8330 U/L), and 1203 U/L (249–21 591 U/L), respectively. Corresponding ferritin levels were 22 236 μg/L (565 to >100 000 μg/L; missing data = 104 patients). Median platelet nadir was 15.5 × 10^9^/L (0–154 × 10^9^/L) and median lowest fibrinogen level was 2.15 g/L (1–5.7 g/L; missing data = 144 patients). The median APACHE II, SAPS II, and SOFA scores within 24 hours of ICU admission were 12, 20, and 6, respectively. With regard to treatment, 70 (39%) patients were intubated, 55 (31%) received inotropic support, and 32 (18%) continuous veno-venous hemofiltration. Twenty-five patients were administered corticosteroids (methylprednisolone alone = 14, dexamethasone alone = 4, hydrocortisone alone = 1, methylprednisolone and dexamethasone together = 6, and intravenous immunoglobulin [IVIG] and methylprednisolone = 2), while none received etoposide. These 25 patients, all with severe organ involvement, were more severely affected, with higher peak AST, ALT, and ferritin levels than those who did not receive corticosteroids (all *P* < .0001). For characteristics of all subgroups see Supplementary Tables S1 and S2.

### Frequency and Clinical Characteristics of Dengue-HLH

Of 180 patients with SD, 77 had 4 or more of the 8 HLH-2004 diagnostic criteria available for evaluation. Of these, 31 (40%) fulfilled 4 or more criteria (group “HLH-2004≥4”), 17% (31 of 180) of all patients with SD (Table 2) [21], while 9 fulfilled 5 or more HLH-2004 criteria. Fifteen of 31 patients had bone marrow evaluation performed; 13 (87%) demonstrated hemophagocytic activity. With regard to HScore, 89 of 180 had sufficient data to evaluate for a probability of HLH of 70% or greater (≥180 points). Of these, 21 (24%) had HLH as defined by HScore (group “HSprobability≥70”), 12% (21 of 180) of all patients with SD (Table 3) [22]. Twelve patients had bone marrow evaluation performed; 11 (92%) revealed hemophagocytosis.

**Table 2.** Presentation of the 180 Patients According to Whether They Fulfilled the Hemophagocytic Lymphohistiocytosis-2004 Diagnostic Criteria or Not or Had <4 Criteria Recorded

	Number of Fulfilled HLH-2004 Criteria ≥4 (n = 31)	Number of Fulfilled HLH-2004 Criteria <4 (n = 46)	Number of Recorded HLH-2004 Criteria <4 (n = 103)
Female	20 (64.5)/11 (35.5)^a^	14 (30.4)/32 (69.6)	43 (41.7)/60 (58.3)
Dead	12 (38.7)/19 (61.3)	11 (23.9)/35 (76.1)	16 (15.5)/87 (84.5)
Splenomegaly	2 (6.7)/28 (93.3); 1	2 (4.4)/43 (95.6); 1	3 (3.9)/73 (96.1); 27
Hepatomegaly	8 (26.7)/22 (73.3); 1	12 (26.7)/33 (73.3); 1	13 (17.1)/63 (82.9); 27
Hemophagocytosis	13 (86.7)/2 (13.3); 16	3 (60.0)/2 (40.0); 41	0 (0.0)/0 (0.0); 103
Leak	19 (61.3)/12 (38.7)	26 (56.5)/20 (43.5)	63 (61.2)/40 (38.8)
Bleed	10 (32.3)/21 (67.7)	14 (30.4)/32 (69.6)	40 (38.8)/63 (61.2)
Severe organ involvement	26 (83.9)/5 (16.1)	31 (67.4)/15 (32.6)	42 (42.4)/57 (57.6); 4
Severe organ involvement (liver)	24 (77.4)/7 (22.6)	20 (43.5)/26 (56.5)	24 (24.5)/74 (75.5); 5
Severe organ involvement (CNS)	6 (19.4)/25 (80.6)	11 (23.9)/35 (76.1)	18 (17.5)/85 (82.5)
Severe organ involvement (heart and other)	11 (35.5)/20 (64.5)	9 (19.6)/37 (80.4)	18 (17.5)/85 (82.5)
Intubation and ventilation	19 (61.3)/12 (38.7)	18 (39.1)/28 (60.9)	33 (32.0)/70 (68.0)
Inotropic support	15 (48.4)/16 (51.6)	15 (32.6)/31 (67.4)	25 (24.3)/78 (75.7)
Continuous veno-venous hemodiafiltration	12 (38.7)/19 (61.3)	10 (21.7)/36 (78.3)	10 (9.7)/93 (90.3)
Corticosteroid treatment	15 (48.4)/16 (51.6)	8 (17.4)/38 (82.6)	2 (1.9)/101 (98.1)
Age at hospital admission, y	36.4 (20.9–77.7)^b^	41.45 (19.3–84.3)	33.7 (18.2–75.3)
Length of hospitalization, d			
Survivors	6.1 (3.1–25.9)	5.9 (2.7–60.1)	6.2 (3.2–37)
Nonsurvivors	3.9 (1.3–15.0)	2.3 (0.5–43.7)	3.3 (0.2–22.1)
Length of ICU, d			
Survivors	3.1 (0.9–13.5)	2.4 (0.3–60)	2.7 (0.2–34.6)
Nonsurvivors	2.2 (0.7–14.5)	2.0 (0.3–43.4)	2.5 (0.1–21.7)
Lowest platelets, ×10^9^/L	14 (1–35)	9 (0–91)	18 (1–154)
Peak triglycerides, mmol/L	2.51 (0.87–8.73); 7	2.25 (0.7–11.2); 19	2.42 (0.97–9.7); 91
Lowest fibrinogen, g/L	1.95 (1–3.2); 13	2.8 (1.3–5.7); 31	1.7 (1.6–2.8); 100
Peak ferritin, μg/L	26 603 (816 to >100 000); 2	18 893 (565 to >100 000); 9	22 236 (3758–83 500); 93
Peak aspartate aminotransferase, U/L	2590 (109–30 649)	760.5 (23–27 520)	300 (22–35 427); 4
Peak alanine aminotransferase, U/L	890 (23–8330)	256 (16–6442)	169.5 (12–4304); 1
Peak lactate dehydrogenase, U/L	2913 (327–19 987)	1366 (267–21 591); 1	824 (249–11 117); 6
Peak creatinine, μmol/L	130 (50–850)	110 (50–350)	90 (20–1440)
APACHE II score	14 (3–41)	11 (5–37)	13 (1–46); 2
SAPS II score	31 (6–85)	21.5 (6–82)	19 (6–106); 2
SOFA score	8 (2–18)	7 (0–19)	5 (1–20); 2

Data from Henter et al [21].

Abbreviations: APACHE, Acute Physiology, Age, Chronic Health Evaluation; CNS, central nervous system; HLH, hemophagocytic lymphohistiocytosis; ICU, intensive care unit; SAPS, Simplified Acute Physiology Score; SOFA, Sequential Organ Failure Assessment.

^a^No. (%) yes/no; no. missing (if applicable).

^b^Median (range); no. missing (if applicable).

**Table 3.** Presentation According to HScore of ≥180 or <180, Corresponding to a Probability of Hemophagocytic Lymphohistiocytosis of ≥70% or <70%, or If Too Few Parameters Were Recorded to Reach a Score of 180

	HScore Probability ≥70% (n = 21)	HScore Probability <70% (n = 68)	Too Few HScore Parameters Recorded (n = 91)
Female	11 (52.4)/10 (47.6)^a^	30 (44.1)/38 (55.9)	36 (39.6)/55 (60.4)
Dead	9 (42.9)/12 (57.1)	17 (25.0)/51 (75.0)	13 (14.3)/78 (85.7)
Splenomegaly	3 (15.8)/16 (84.2); 2	1 (1.6)/63 (98.4); 4	3 (4.4)/65 (95.6); 23
Hepatomegaly	9 (47.4)/10 (52.6); 2	12 (18.8)/52 (81.3); 4	12 (17.6)/56 (82.4); 23
Hemophagocytosis	11 (91.7)/1 (8.3); 9	5 (62.5)/3 (37.5); 60	0 (0.0)/0 (0.0); 91
Leak	13 (61.9)/8 (38.1)	39 (57.4)/29 (42.6)	56 (61.5)/35 (38.5)
Bleed	7 (33.3)/14 (66.7)	21 (30.9)/47 (69.1)	36 (39.6)/55 (60.4)
Severe organ involvement	18 (85.7)/3 (14.3)	44 (64.7)/24 (35.3)	37 (42.5)/50 (57.5); 4
Severe organ involvement (liver)	18 (85.7)/3 (14.3)	30 (44.1)/38 (55.9)	20 (23.3)/66 (76.7); 5
Severe organ involvement (CNS)	5 (23.8)/16 (76.2)	14 (20.6)/54 (79.4)	16 (17.6)/75 (82.4)
Severe organ involvement (heart and other)	6 (28.6)/15 (71.4)	17 (25.0)/51 (75.0)	15 (16.5)/76 (83.5)
Intubation and ventilation	14 (66.7)/7 (33.3)	28 (41.2)/40 (58.8)	28 (30.8)/63 (69.2)
Inotropic support	11 (52.4)/10 (47.6)	23 (33.8)/45 (66.2)	21 (23.1)/70 (76.9)
Continuous veno-venous hemodiafiltration	9 (42.9)/12 (57.1)	14 (20.6)/54 (79.4)	9 (9.9)/82 (90.1)
Corticosteroid treatment	14 (66.7)/7 (33.3)	11 (16.2) / 57 (83.8)	0 (0.0)/91 (100.0)
Age at hospital admission, y	33.5 (19.3–51.2)^b^	41.05 (18.9–84.3)	32.6 (18.2–75.3)
Length of hospitalization, d			
Survivors	6.1 (3.1–60.1)	6.1 (2.7–24.2)	6.1 (3.2–37)
Nonsurvivors	3.1 (1.3–15)	1.9 (0.2–43.7)	4.3 (0.9–22.1)
Length of ICU, d			
Survivors	3.7 (1.7–60)	2.7 (0.3–8.1)	2.7 (0.2–34.6)
Nonsurvivors	1.6 (0.7–14.5)	1.3 (0.1–43.4)	4.3 (0.2–21.7)
Lowest platelets, ×10^9^/L	15 (1–35)	9.5 (0–95)	21 (1–154)
Peak triglycerides, mmol/L	2.825 (1.3–6.94); 1	2.23 (0.7–11.2); 26	9.7 (9.7–9.7); 90
Lowest fibrinogen, g/L	1.9 (1–2.9); 6	2.8 (1–5.7); 49	2.2 (1.6–2.8); 89
Peak ferritin, μg/L	29 531 (2420 to >100 000)	20 989 (565 to >100 000); 18	7319 (3758–44 554); 86
Peak aspartate aminotransferase, U/L	2858 (265–30 189)	760.5 (23–30 649)	288 (22–35 427); 4
Peak alanine aminotransferase, U/L	890 (105–6081)	266.5 (16–8330)	166 (12–3790); 1
Peak lactate dehydrogenase, U/L	4830 (327–19 987)	1321 (267–21 591); 1	825 (249–11 117); 6
Peak creatinine, μmol/L	220 (50–850)	102 (40–1440)	90 (20–810)
APACHE II score	16 (3–37)	11 (5–41); 2	12 (1–46)
SAPS II score	32 (6–73)	21.5 (6–94); 2	18 (6–106)
SOFA score	8 (5–16)	7 (0–19); 2	5 (1–20)

Data from Fardet et al [22].

Abbreviations: APACHE, Acute Physiology, Age, Chronic Health Evaluation; CNS, central nervous system; HLH, hemophagocytic lymphohistiocytosis; HScore, histo score; ICU, intensive care unit; SAPS, Simplified Acute Physiology Score; SOFA, Sequential Organ Failure Assessment.

^a^No. (%) yes/no; no. missing (if applicable).

^b^Median (range); no. missing (if applicable).

Median nadir platelet count, lowest fibrinogen level, and median peak AST, ALT, LDH, and ferritin levels in HLH-2004≥4criteria patients and among HSprobability≥70 patients are presented in Tables 2 and 3, as well as median APACHE II, SAPS II, and SOFA scores. Four patients with SD were also immunosuppressed, but only 1 patient, with lupus nephritis treated with azathioprine and prednisolone on admission, had an HScore probability of HLH of 70% or greater.

Clinical and laboratory findings for the 180 patients with SD associated with having HLH, defined as an HScore probability of HLH of 70% or greater, included hepatomegaly (OR, 3.9; *P* = .015), peak AST (log U/L) (OR, 1.6; *P* = .0043), peak ALT (log U/L) (OR, 1.7; *P* = .0075), peak LDH (log U/L) (OR, 1.8; *P* = .010), and peak ferritin (log μg/L) (OR, 1.7; *P* = .048) levels, while increasing age at hospitalization (by 5-year increments) was negatively associated (OR, 0.8; *P* = .036) (Table 4). The corresponding findings for having 4 or more HLH-2004 criteria were female gender (OR, 4.2; *P* = .0039), peak AST (log U/L) (OR, 1.5; *P* = .017), peak ALT (log U/L) (OR, 1.5; *P* = .024), peak LDH (log U/L) (OR, 1.65; *P* = .024), and lowest fibrinogen (log g/L) (OR, 8.35; *P* = .040) levels (Table 4).

**Table 4.** Odds Ratios for Clinical and Laboratory Findings of an HScore Probability of Hemophagocytic Lymphohistiocytosis (HLH) ≥70% and of ≥4 HLH-2004 Criteria Fulfilled

Covariate	n	OR for HScore Probability≥70% (95% CI; *P*)^a^	n	OR for ≥4 HLH-2004 Criteria (95% CI; *P* Value)^a^
Female gender	89	1.39 (0.52–3.72; .51)	77	4.16 (1.58–10.93; .0039)
Severe bleed	89	1.12 (0.39–3.18; .83)	77	1.09 (0.41–2.90; .87)
Severe leak	89	1.21 (0.44–3.30; .71)	77	1.22 (0.48–3.08; .68)
Severe organ involvement	89	3.27 (0.87–12.25; .078)	77	2.52 (0.81–7.86; .11)
Hepatomegaly	83	3.90 (1.30–11.69; .015)	75	1.00 (0.35–2.84; 1.0)
Age at hospitalization (5 years)^b^	89	0.79 (0.63–0.98; .036)	77	0.98 (0.83–1.14; .76)
APACHE II score^c^	87	1.03 (0.98–1.08; .29)	77	1.01 (0.97–1.06; .57)
SAPS II score^c^	87	1.01 (0.99–1.03; .30)	77	1.01 (0.99–1.03; .37)
SOFA score^c^	87	1.08 (0.97–1.20; .14)	77	1.03 (0.93–1.13; .60)
Peak aspartate aminotransferase (U/L)^d^	89	1.63 (1.17–2.28; .0043)	77	1.46 (1.07–1.98; .017)
Peak alanine aminotransferase (U/L)^d^	89	1.69 (1.15–2.48; .0075)	77	1.49 (1.05–2.10; .024)
Peak lactate dehydrogenase (U/L)^d^	88	1.84 (1.15–2.92; .010)	76	1.65 (1.07–2.54; .024)
Peak ferritin (log μg/L)^d^	71	1.68 (1.01–2.82; .048)	66	1.52 (0.97–2.39; .070)
Peak triglycerides (log mmol/L)^d^	62	2.62 (0.97–7.04; .057)	51	1.16 (0.45–2.95; .76)
Lowest fibrinogen (log g/L)^e^	34	5.95 (0.88–40.36; .068)	33	8.35 (1.10–63.54; .040)
Lowest platelets (log ×10^9^/L)^e^	88	0.95 (0.59–1.54; .84)	76	1.13 (0.72–1.77; .59)
Peak creatinine (log μmol/L)^d^	89	1.92 (0.96–3.84; .067)	77	1.42 (0.70–2.90; .33)

Abbreviations: APACHE, Acute Physiology, Age, Chronic Health Evaluation; CI, confidence interval; HLH, hemophagocytic lymphohistiocytosis; HScore, histo score; OR, odds ratio; SAPS, Simplified Acute Physiology Score; SOFA, Sequential Organ Failure Assessment.

^a^Calculated using logistic regression.

^b^OR for a 5-year increase.

^c^OR for a 1-unit increase.

^d^OR for a 1-unit increase in the natural logarithm.

^e^OR for a 1-unit decrease in the natural logarithm.

Complications among the 31 patients with 4 or more HLH-2004 criteria included the following: ICU sepsis (n = 5), peptic ulcer (n = 5), acute respiratory distress syndrome (n = 4), myocarditis (n = 3), arrhythmia (n = 3), nasal-oral bleed (n = 3), acute pulmonary edema (n = 2), hematoma (n = 1), vocal cord palsy (n = 1), compartment syndrome (n = 1), and bleeding from bone marrow site (n = 1). Interventions performed included, for example, esophago-gastric-duodenoscopy, laparotomy due to ruptured corpus luteal cyst, and wound debridement.

### Mortality of Severe Dengue and Dengue-HLH

Altogether 39 of 180 (22%) patients with SD died, including 12 of 31 (39%) HLH-2004≥4criteria and 9 of 21 (43%) HSprobability≥70 patients. The fatality rate among patients with severe leak was 29 of 108 (27%), with severe bleed was 23 of 64 (36%), and with severe organ involvement (liver, CNS, and heart and other) was 37 of 99 (37%) (Supplementary Table S1). Specifically, mortality was 32 of 68 (47%) patients with severe liver involvement. Notably, 36 of 39 (92%) who died were of working age (18–65 years; 16 men). Altogether, 13 of 25 (52%) patients who received corticosteroids survived, including 8 of 10 (80%) who were administered dexamethasone. Among HLH-2004≥4criteria patients 7 of 15 and among HSprobability≥70 patients 6 of 14 who received corticosteroids survived.

**Figure 1.**
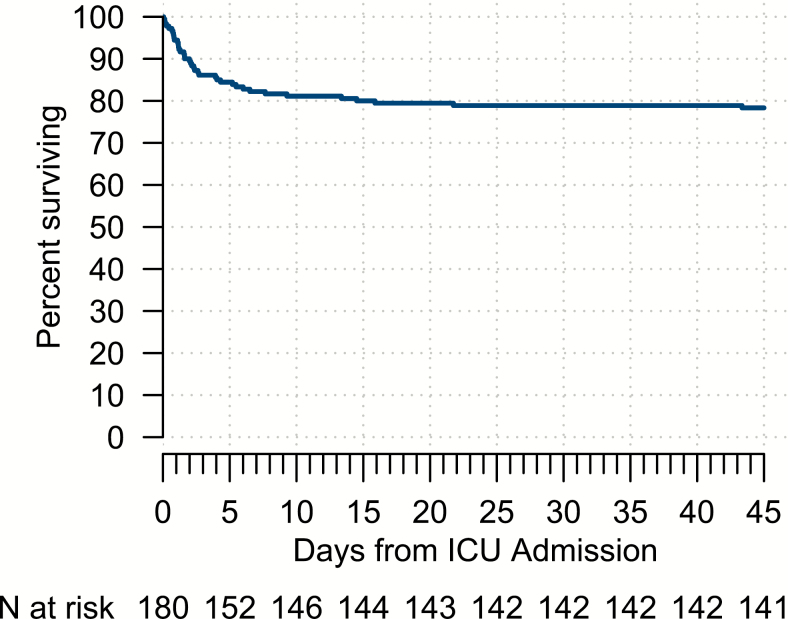
Survival curve. Kaplan-Meier survival estimates in relation to days from ICU admission for all adult patients with severe dengue admitted to ICU (N = 180); the probability of survival from day 43 was 78%. Abbreviation: ICU, intensive care unit.

The median duration for all nonsurvivors from the onset of dengue symptoms to death was 7 days (interquartile range [IQR], 6–12; range, 4–45 days), but the median duration from ICU admission to death was only 2 days (IQR, 1–5; range: 0–43 days), with 25 of 39 (64%) deaths occurring during the first 3 days of ICU admission (Figure 1).

### Parameters Related to Mortality

Clinical and laboratory findings in the 141 survivors and 39 nonsurvivors are detailed in Table 1. Peak values of AST, ALT, LDH, ferritin, and creatinine were all markedly higher in nonsurvivors, and platelet and fibrinogen nadir markedly lower. Furthermore, as expected, APACHE II, SAPS II, and SOFA scores within 24 hours of ICU admission were all markedly higher in nonsurvivors. Mortality in relation to various levels of ferritin and AST is presented in Supplementary Table S3.

In univariable logistic regression, severe organ involvement (OR, 23.6; *P* < .0001), severe bleed (OR, 3.5; *P* = .00081), and severe leak (OR, 2.3; *P* = .042) all correlated with increased risk of death (Table 5). Peak AST, ALT, and LDH (log U/L) all strongly correlated with fatality (ORs, 2.9, 3.4 and 5.8, respectively; all *P* < .0001). Peak ferritin (log μg/L) (OR, 2.5; *P* = .0028), peak creatinine (OR, 32; *P* < .0001), lowest platelet levels (log × 10^9^/L) (OR, 1.9; *P* = .00068), and hepatomegaly (OR, 2.9; *P* = .012) were also mortality-associated risk factors, as was increasing age at hospitalization (by 5-year increment) (OR, 1.2; *P* = .0043). Finally, APACHE II, SAPS II, and SOFA scores all related to fatality (ORs, 1.3, 1.1 and 1.8, respectively; *P* < .0001 for all) (Table 5). Notably, increasing age was also positively correlated with these scores (data not shown).

**Table 5.** Univariable Logistic Regression for Death in Patients With Severe Dengue

Covariate	n	OR (95% CI; *P* Value)^a^
Female gender	180	1.77 (0.87–3.62; .12)
Severe bleed	180	3.51 (1.68–7.31; .00081)
Severe leak	180	2.28 (1.03–5.02; .042)
Severe organ involvement	180	23.57 (5.47–101.62; <.0001)
Hepatomegaly	151	2.89 (1.27–6.57; .012)
Age at hospitalization (5 years)^b^	180	1.20 (1.06–1.35; .0043)
APACHE II score^c^	178	1.33 (1.21–1.45; <.0001)
SAPS II score^c^	178	1.15 (1.10–1.20; <.0001)
SOFA score^c^	178	1.84 (1.53–2.21; <.0001)
Peak aspartate aminotransferase (U/L)^d^	176	2.92 (2.07–4.12; <.0001)
Peak alanine aminotransferase (U/L)^d^	179	3.36 (2.28–4.95; <.0001)
Peak lactate dehydrogenase (U/L)^d^	173	5.80 (3.37–9.98; <.0001)
Peak ferritin (log μg/L)^d^	76	2.54 (1.38–4.68; .0028)
Peak triglycerides (log mmol/L)^d^	63	0.73 (0.29–1.86; .51)
Lowest fibrinogen (log g/L)^e^	36	3.34 (0.53–21.03; .20)
Lowest platelets (log ×10^9^/L)^e^	179	1.86 (1.30–2.65; .00068)
Peak creatinine (log μmol/L)^d^	180	31.93 (11.21–91.01; <.0001)

Abbreviations: APACHE, Acute Physiology, Age, Chronic Health Evaluation; CI, confidence interval; OR, odds ratio; SAPS, Simplified Acute Physiology Score; SOFA, Sequential Organ Failure Assessment.

^a^Calculated using logistic regression.

^b^OR for a 5-year increase.

^c^OR for a 1-unit increase.

^d^OR for a 1-unit increase in the natural logarithm.

^e^OR for a 1-unit decrease in the natural logarithm.

**Figure 2.**
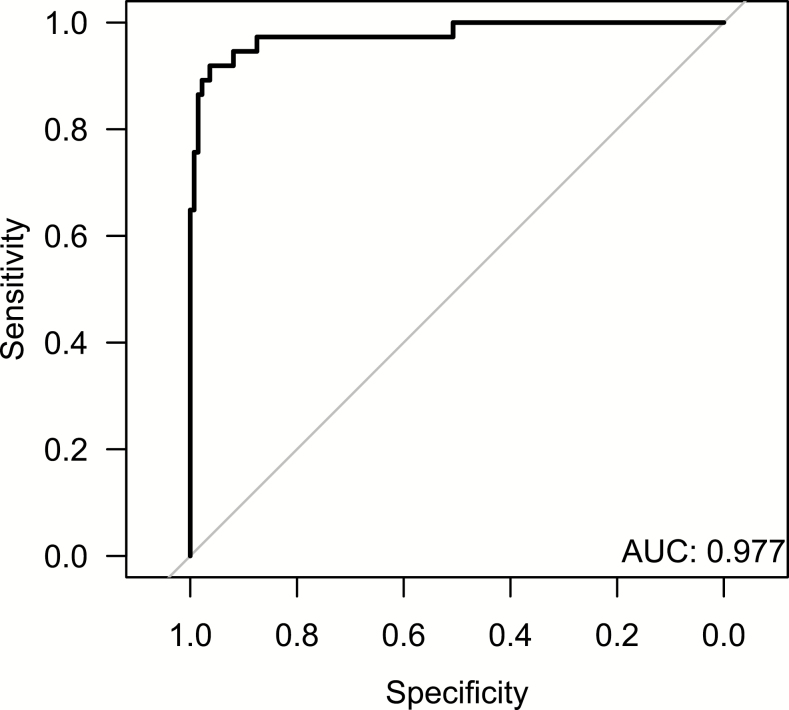
Receiver operating characteristic curve for the multivariable logistic regression model, including the variables age at hospitalization, SOFA score, peak aspartate aminotransferase, and peak creatinine. Abbreviations: AUC, area under the curve; SOFA, Sequential Organ Failure Assessment.

In the multivariable logistic regression model, peak AST (log U/L) (OR, 2.8; *P* = .0019), peak creatinine (OR, 7.3; *P* = .0065), and SOFA score (OR, 1.4; *P* = .0051) turned out to be independent risk factors for death (Table 6). The model had an AUC of 0.98 (Figure 2). Using a cutoff for the log-odds of 0.33 resulted in a sensitivity of 92% and a specificity of 96%.

**Table 6.** Multivariable Logistic Regression for Death in Patients With Severe Dengue

Covariate	n	OR (95% CI; *P* Value)^a^
Age at hospitalization^b^	173	1.33 (0.99–1.79; .058)
SOFA score^c^	173	1.41 (1.11–1.79; .0051)
Peak aspartate aminotransferase (log U/L)^d^	173	2.76 (1.45–5.24; .0019)
Peak creatinine (log μl/L)^d^	173	7.34 (1.75–30.78; .0065)

The model intercept was given by −24.57 (−35.44 to −13.69). Abbreviations: CI, confidence interval; OR, odds ratio; SOFA, Sequential Organ Failure Assessment.

^a^Calculated using logistic regression.

^b^OR for a 5-year increase.

^c^OR for a 1-unit increase.

^d^OR for a 1-unit increase in the natural logarithm.

## DISCUSSION

Dengue cases have increased 30-fold in the past 50 years [20]. In our cohort, mortality in SD was 22% (39 of 180) and, notably, was ~40% in dengue-HLH. Peak values of AST, ALT, LDH, ferritin, and creatinine were mortality-associated risk factors, as were lowest platelet counts, hepatomegaly, severe organ involvement, severe bleed, severe leak, increasing age at hospitalization, and APACHE II, SAPS II, and SOFA scores (Table 5). Due to its high mortality, it is important to be observant of dengue-HLH and to consider HLH-directed therapy in affected patients.

To our knowledge, this report is the largest and most comprehensive on dengue-HLH in adults so far [11–18], except for a recent meta-analysis [19]. In children, there are 2 large studies on dengue-HLH; 1 from India reporting 23 children with HLH (≥5 of 8 fulfilled HLH-2004 criteria, including bone marrow hemophagocytosis) of 212 children with dengue infection [14] and 1 from Puerto Rico on 22 children who fulfilled ≥5 of 8 HLH-2004 criteria [17]. These reports, and the meta-analysis, support that HLH should be considered in rapidly deteriorating patients with SD with persistent fever, cytopenia, and markedly elevated ferritin and liver enzyme levels. Notably, ferritin has been reported to be valuable in identifying critically ill patients with sHLH, and even specifically predicting SD [27–29].

We found that the high dengue-HLH mortality makes it reasonable to consider a short course of HLH-directed therapy in selected patients. In line, prednisolone use has been significantly associated with less derangement in leukocyte and AST levels (*P* < .001 and *P* = .01, respectively) [30]. Importantly, HLH-directed treatment with dexamethasone and etoposide showed substantially reduced mortality in another potentially fatal viral infection associated with HLH (ie, severe EBV-HLH) [8–10]. Notably, T cells are infected in both EBV-HLH and acutely infected patients with dengue, and in dengue infection, T cells support viral replication and secrete viable virus particles. HLH-directed treatment efficacy in EBV-HLH may partly be due to lymphocyte reduction by corticosteroids and etoposide, which provides a rationale for a similar treatment approach in dengue-HLH [31, 32]. Etoposide has been instrumental in reducing mortality in primary and secondary HLH [5–10]. A French study from a medical ICU concluded that there is a risk of missing the time frame when HLH-directed treatment may be effective [33]. In another study in 162 patients with HLH, etoposide included in first-line treatment tended to result in a better outcome (*P* = .079) [34]. In the dengue-HLH study from Puerto Rico, 16 of 22 (73%) patients were administered corticosteroids, 13 (59%) IVIG, 8 (36%) etoposide, and 8 (36%) “chemotherapy” (not defined further) [17]. Since only 1 child died, survival in etoposide-treated dengue-HLH was 7 of 8 (88%) or 8 of 8 (100%). A PubMed search on 15 April 2019 on “dengue, etoposide” revealed 2 additional reports where etoposide may have been used in dengue-HLH, but without specifics on outcome [35, 36]. The exact mechanism of etoposide in hyperinflammation treatment is not established, but etoposide has been shown to substantially alleviate all symptoms of murine HLH, and the pharmacodynamics involved potent selective deletion of activated T cells and efficient suppression of inflammatory cytokine production [37].

Of note, patients treated with corticosteroids in our study had more severe disease (higher peak AST, ALT, and ferritin levels; *P* < .0001). Nevertheless, 8 of 10 treated with dexamethasone survived. While these numbers are small, they are at least indicative and together with literature data suggest that early use of steroids in the context of HLH is worth further studies in SD [17, 30], possibly with the addition of etoposide in patients with more pronounced hyperinflammation or rapidly deteriorating status, in particular patients with severe liver involvement (47% mortality). In our study, 25 of 39 (64%) of those who died, died within 3 days of ICU admission. Furthermore, they also manifested significantly higher levels of AST, ALT, LDH, creatinine, and ferritin than did survivors, which was particularly noticeable in dengue-HLH. Increasing peak ferritin and AST levels were associated with increasing fatality. In patients with dengue and with persistent fever and cytopenia along with severe organ involvement, a drastic increase in liver enzymes and/or altered mental state, in particular if ferritin is greater than 10 000 μg/L, clinicians need to consider HLH. Moreover, in infection-associated HLH in adults the etoposide dose is suggested to be reduced as compared with HLH-94/HLH-2004, from 150 mg/m^2^ intravenous (iv) per dose to 50–100 mg/m^2^ iv administered once weekly and only for a short period [38].

Our study has several limitations. First, patients may have died of SD and dengue-HLH in the region without being diagnosed (ie, fatalities may have been underestimated). Second, corticosteroid efficacy cannot be evaluated because it was not used in a structured manner. Third, laboratory results for ferritin, AST, ALT, and LDH were sometimes so high that diluted samples would have been necessary to obtain correct values (ie, sometimes the peak values reported were falsely low). Similarly, infrequent sampling may have resulted in missing peak and nadir levels. Fourth, making a diagnosis of secondary HLH is difficult. Fulfilment of 5 or more of 8 HLH-2004 criteria serves as a practical tool for HLH diagnosis [21], but these criteria were developed for children and are not validated formally for adults. In this study, 2 of 8 HLH-2004 criteria (NK-cell activity; soluble CD25) were not evaluated in any patient. Moreover, 3 additional criteria were evaluated in fewer than half of patients (hemophagocytosis, ferritin, hypertriglyceridemia/hypofibrinogenemia). Due to these limitations, we report both fulfillment of 4 or more and 5 or more of 8 HLH-2004 criteria [21], in addition to evaluation by HScore (retrospectively developed in a selected cohort of 312 adults) [22]. The reported best cutoff value for HScore was 169 points (~55% probability of HLH), corresponding to an accurate classification of 90% of patients. We used a higher level for probability of HLH (≥70%), to reduce overestimation of HLH. The frequent lack of diagnostic HLH parameters is an important study limitation. However, with more diagnostic parameters studied, the proportion of dengue-HLH could be even higher.

We conclude that it is important to be aware of dengue-HLH in SD due to its high mortality. Furthermore, a prospective study is needed in patients with, or at high risk of developing, dengue-HLH to evaluate prompt HLH-directed therapy with corticosteroids such as dexamethasone, and in very severe cases the possible addition of etoposide, as a complement to standard supportive management.

## Supplementary Data

Supplementary materials are available at *Clinical Infectious Diseases* online. Consisting of data provided by the authors to benefit the reader, the posted materials are not copyedited and are the sole responsibility of the authors, so questions or comments should be addressed to the corresponding author.

ciz499_suppl_Supplementary_Table_S1

ciz499_suppl_Supplementary_Table_S2

ciz499_suppl_Supplementary_Table_S3

## Notes

***Author contributions.*** F. K. K. and C. C. T. provided study concept, methodology, data, investigations and analyses; C. C. T. also provided resources and F. F. K. administered the study. T. v. B. G. interpreted data and contributed to drafting the manuscript. K. E. K. provided data and investigations. P. S. performed statistical analyses. I. H. M. analyzed data and performed statistical analyses. L. H. T. conceptualized and supervised the study. J.-I. H. analyzed data, interpreted data, and drafted the manuscript, which was reviewed by all authors.

***Disclaimer.*** The funding sources had no involvement in the study.

***Financial support.*** This work was supported by the Stockholm County Council (ALF project) (grant number 20150016; to J.-I. H.), the Swedish Children’s Cancer Foundation (grant numbers PROF06/001 and PR2015/005; to J.-I. H.), and the Swedish Cancer Foundation (grant number CAN 2016/275 ; to J.-I.H.).

***Potential conflicts of interest.*** The authors: No reported conflicts of interest. All authors have submitted the ICMJE Form for Disclosure of Potential Conflicts of Interest. Conflicts that the editors consider relevant to the content of the manuscript have been disclosed.
