# Supplementary material for: Dengue Infection Complicated by Hemophagocytic Lymphohistiocytosis: Experiences From 180 Patients With Severe Dengue
Source: Clin Infect Dis. 2019 Jun 12;70(11):2247–55. doi: 10.1093/cid/ciz499 (PMC7245144; doi:10.1093/cid/ciz499)
Supplement: ciz499_suppl_Supplementary_Table_S1 [file ciz499_suppl_supplementary_table_s1.docx]

**Supplementary Table S1:**

**Presentation of all the 180 patients with severe dengue and the subgroups severe leak, severe bleed, and severe organ involvement.**

|  | **All (n=180)** | **Severe leak (n=108)** | **Severe bleed (n=64)** | **Severe organ involvement: Liver, CNS or "Heart and Other" (n=99)** |
| --- | --- | --- | --- | --- |
|  | n Yes (%) / n No (%) / n Missing | |  |  |
| Female | 77 (42.8) / 103 (57.2) | 48 (44.4) / 60 (55.6) | 27 (42.2) / 37 (57.8) | 46 (46.5) / 53 (53.5) |
| Dead | 39 (21.7) / 141 (78.3) | 29 (26.9) / 79 (73.1) | 23 (35.9) / 41 (64.1) | 37 (37.4) / 62 (62.6) |
| Splenomegaly | 7 (4.6) / 144 (95.4) / 29 | 4 (4.4) / 86 (95.6) / 18 | 4 (7.5) / 49 (92.5) / 11 | 6 (6.9) / 81 (93.1) / 12 |
| Hepatomegaly | 33 (21.9) / 118 (78.1) / 29 | 19 (21.1) / 71 (78.9) / 18 | 11 (20.8) / 42 (79.2) / 11 | 23 (26.4) / 64 (73.6) / 12 |
| Hemophagocytosis | 16 (80.0) / 4 (20.0) / 160 | 13 (81.3) / 3 (18.8) / 92 | 6 (75.0) / 2 (25.0) / 56 | 16 (80.0) / 4 (20.0) / 79 |
| Leak | 108 (60.0) / 72 (40.0) | 108 (100.0) / 0 (0.0) | 29 (45.3) / 35 (54.7) | 51 (51.5) / 48 (48.5) |
| Bleed | 64 (35.6) / 116 (64.4) | 29 (26.9) / 79 (73.1) | 64 (100.0) / 0 (0.0) | 37 (37.4) / 62 (62.6) |
| Severe organ involvement | 99 (56.3) / 77 (43.8) / 4 | 51 (48.6) / 54 (51.4) / 3 | 37 (58.7) / 26 (41.3) / 1 | 99 (100.0) / 0 (0.0) |
| Severe organ involvement (Liver) | 68 (38.9) / 107 (61.1) / 5 | 41 (39.4) / 63 (60.6) / 4 | 28 (44.4) / 35 (55.6) / 1 | 68 (69.4) / 30 (30.6) / 1 |
| Severe organ involvement (CNS) | 35 (19.4) / 145 (80.6) | 11 (10.2) / 97 (89.8) | 10 (15.6) / 54 (84.4) | 35 (35.4) / 64 (64.6) |
| Severe organ involvement (Heart and Other) | 38 (21.1) / 142 (78.9) | 26 (24.1) / 82 (75.9) | 21 (32.8) / 43 (67.2) | 38 (38.4) / 61 (61.6) |
| Intubation and ventilation | 70 (38.9) / 110 (61.1) | 47 (43.5) / 61 (56.5) | 36 (56.3) / 28 (43.8) | 62 (62.6) / 37 (37.4) |
| Inotropic support | 55 (30.6) / 125 (69.4) | 41 (38.0) / 67 (62.0) | 34 (53.1) / 30 (46.9) | 50 (50.5) / 49 (49.5) |
| Continuous veno-venous hemodiafiltration | 32 (17.8) / 148 (82.2) | 28 (25.9) / 80 (74.1) | 20 (31.3) / 44 (68.8) | 30 (30.3) / 69 (69.7) |
| Corticosteroid treatment | 25 (13.9) / 155 (86.1) | 18 (16.7) / 90 (83.3) | 10 (15.6) / 54 (84.4) | 25 (25.3) / 74 (74.7) |
|  | Median (range), n Missing |  |  |  |
| Age at hospital admission (years) | 34.9 (18.2 - 84.3) | 34.4 (18.6 - 84.3) | 41.5 (18.9 - 79.7) | 40.4 (18.2 - 84.3) |
| Length of hospitalization (days) (survivors) | 6 .1 (2.7 - 60.1) | 6.1 (2.7 - 60.1) | 6.9 (3.6 - 60.1) | 7.1 (3.1 - 60.1) |
| Length of hospitalization (days) (non-survivors) | 2 .6 (0.2 - 43.7) | 2.3 (0.2 - 43.7) | 2.4 (0.5 - 22.1) | 2.6 (0.2 - 43.7) |
| Length of ICU (days) (survivors) | 2.7 (0.2 - 60.0) | 2.8 (0.4 - 60) | 3.5 (0.6 - 60.0) | 3.2 (0.2 - 60.0) |
| Length of ICU (days) (non-survivors) | 2.1 (0.1 - 43.3) | 1.6 (0.1 - 43.4) | 2.2 (0.3 - 21.7) | 2.1 (0.1 - 43.4) |
| Lowest platelets (x10^9^/L) | 15.5 (0 - 154) | 13.5 (0 - 120) | 17 (1 - 154) | 14 (0 - 133) |
| Peak triglycerides (mmol/L) | 2.33 (0.7 - 11.2), 117 | 2.35 (0.7 - 11.2), 69 | 2.665 (1.11 - 9.7), 46 | 2.14 (0.7 - 6.94), 56 |
| Lowest fibrinogen (g/L) | 2.15 (1 - 5.7), 144 | 2.3 (1 - 5.7), 87 | 1.55 (1 - 3.2), 54 | 2.1 (1 - 5.7), 70 |
| Peak ferritin (microg/L) | 22 236 (565 - >100 000), 104 | 24 114 (2 420 - >100 000), 69 | 20 665 (565 - >100 000), 39 | 27 922 (816 - >100 000), 44 |
| Peak aspartate aminotransferase (U/L) | 510 (22 - 35 427), 4 | 505 (35 - 35 427), 3 | 773 (22 - 35 427), 1 | 1 763 (22 - 35 427) |
| Peak alanine aminotransferase (U/L) | 246 (12 - 8 330), 1 | 282 (12 - 8 330), 1 | 256 (16 - 6 442) | 761 (23 - 8 330), 1 |
| Peak lactate dehydrogenase (U/L) | 1 203 (249 - 21 591), 7 | 1 250 (249 - 21 591), 5 | 1 333.5 (267 - 15 664), 2 | 2 384.5 (269 - 21 591), 1 |
| Peak creatinine (micromol/L) | 100 (20 - 1 440) | 110 (20 - 1 440) | 130 (30 - 610) | 140 (30 - 1 440) |
| APACHE II score | 12 (1 - 46), 2 | 13 (2 - 41), 2 | 16 (1 - 46) | 17 (3 - 45), 1 |
| SAPS II score | 20 (6 - 106), 2 | 20 (6 - 94), 2 | 29 (6 - 106) | 33 (6 - 103), 1 |
| SOFA score | 6 (0 - 20), 2 | 6.5 (1 - 19), 2 | 8 (1 - 20) | 8 (0 - 20), 1 |
