# Supplementary material for: Dengue Infection Complicated by Hemophagocytic Lymphohistiocytosis: Experiences From 180 Patients With Severe Dengue
Source: Clin Infect Dis. 2019 Jun 12;70(11):2247–55. doi: 10.1093/cid/ciz499 (PMC7245144; doi:10.1093/cid/ciz499)
Supplement: ciz499_suppl_Supplementary_Table_S2 [file ciz499_suppl_supplementary_table_s2.docx]

**Supplementary Table S2:**

**Presentation of the subgroups severe organ involvement of the liver, of the CNS, and of the heart and other organs.**

|  | **Severe organ involvement:**  **Liver (n=68)** | **Severe organ involvement: CNS (n=35)** | **Severe organ involvement: Heart and other (n=38)** |
| --- | --- | --- | --- |
|  | n Yes (%) / n No (%) / n Missing |  |  |
| Female | 33 (48.5) / 35 (51.5) | 13 (37.1) / 22 (62.9) | 20 (52.6) / 18 (47.4) |
| Dead | 32 (47.1) / 36 (52.9) | 10 (28.6) / 25 (71.4) | 23 (60.5) / 15 (39.5) |
| Splenomegaly | 5 (8.2) / 56 (91.8) / 7 | 1 (3.4) / 28 (96.6) / 6 | 3 (8.3) / 33 (91.7) / 2 |
| Hepatomegaly | 21 (34.4) / 40 (65.6) / 7 | 6 (20.7) / 23 (79.3) / 6 | 6 (16.7) / 30 (83.3) / 2 |
| Hemophagocytosis | 16 (84.2) / 3 (15.8) / 49 | 4 (11.4) / 31 (88.6) | 9 (90.0) / 1 (10.0) / 28 |
| Leak | 41 (60.3) / 27 (39.7) | 11 (31.4) / 24 (68.6) | 26 (68.4) / 12 (31.6) |
| Bleed | 28 (41.2) / 40 (58.8) | 10 (28.6) / 25 (71.4) | 21 (55.3) / 17 (44.7) |
| Severe organ involvement (Liver) | 68 (100.0) / 0 (0.0) | 16 (47.1) / 18 (52.9) / 1 | 23 (62.2) / 14 (37.8) / 1 |
| Severe organ involvement (CNS) | 16 (23.5) / 52 (76.5) | 35 (100.0) / 0 (0.0) | 9 (23.7) / 29 (76.3) |
| Severe organ involvement (Heart and Other) | 23 (33.8) / 45 (66.2) | 9 (25.7) / 26 (74.3) | 38 (100.0) / 0 (0.0) |
| Intubation and ventilation | 43 (63.2) / 25 (36.8) | 22 (62.9) / 13 (37.1) | 33 (86.8) / 5 (13.2) |
| Inotropic support | 36 (52.9) / 32 (47.1) | 14 (40.0) / 21 (60.0) | 32 (84.2) / 6 (15.8) |
| Continuous veno-venous hemodiafiltration | 28 (41.2) / 40 (58.8) | 8 (22.9) / 27 (77.1) | 18 (47.4) / 20 (52.6) |
| Corticosteroid treatment | 24 (35.3) / 44 (64.7) | 7 (20.0) / 28 (80.0) | 11 (28.9) / 27 (71.1) |
|  | Median (range), n Missing |  |  |
| Age at hospital admission (years) | 41.45 (19.3 - 84.3) | 38.3 (20.9 - 75.3) | 44.65 (18.2 - 84.3) |
| Length of hospitalization (days) (survivors) | 7.1 (3.1 - 60.1) | 8.0 (3.2 - 34.7) | 7.9 (4.0 - 25.9) |
| Length of hospitalization (days) (non-survivors) | 2.4 (0.2 - 22.1) | 7.2 (0.9 - 43.7) | 2.4 (0.2 - 22.1) |
| Length of ICU (days) (survivors) | 3.4 (0.3 - 60.0) | 3.1 (0.2 - 7.8) | 4.1 (1.9 - 15.6) |
| Length of ICU (days) (non-survivors) | 2.0 (0.1 - 21.7) | 5.7 (0.2 - 43.4) | 2.0 (0.1 - 21.7) |
| Lowest platelets (x10^9^/L) | 10 (0 - 95) | 18 (1 - 133) | 14 (3 - 99) |
| Peak triglycerides (mmol/L) | 2.08 (0.7 - 6.94), 33 | 1.705 (0.97 - 6.16), 21 | 2.065 (0.7 - 6.94), 22 |
| Lowest fibrinogen (g/L) | 1.75 (1 - 5), 46 | 2.5 (1.3 - 5.7), 25 | 1.8 (1 - 5), 27 |
| Peak ferritin (microg/L) | 31 648.5 (2 420 - >100 000), 26 | 25 306 (816 - >100 000), 18 | 36 548 (5 798 - >100 000), 21 |
| Peak aspartate aminotransferase (U/L) | 2 884 (533 - 35 427) | 816 (128 - 35 427) | 1 310.5 (22 - 30 649) |
| Peak alanine aminotransferase (U/L) | 1 501 (105 - 8 330) | 306 (67 - 4 304), 1 | 534 (23 - 8 330), 1 |
| Peak lactate dehydrogenase (U/L) | 3 846 (327 - 21 591), 1 | 1 429 (327 - 12 588) | 2 131 (269 - 21 591) |
| Peak creatinine (micromol/L) | 180 (40 - 1 440) | 120 (30 - 850) | 250 (40 - 1 440) |
| APACHE II score | 17 (3 - 45), 1 | 16 (7 - 45) | 22 (6 - 45), 1 |
| SAPS II score | 33 (6 - 103), 1 | 34 (9 - 103) | 48 (10 - 103), 1 |
| SOFA score | 8 (4 - 20), 1 | 8 (0 - 20) | 13 (4 - 20), 1 |
