# Supplementary material for: Dengue Infection Complicated by Hemophagocytic Lymphohistiocytosis: Experiences From 180 Patients With Severe Dengue
Source: Clin Infect Dis. 2019 Jun 12;70(11):2247–55. doi: 10.1093/cid/ciz499 (PMC7245144; doi:10.1093/cid/ciz499)
Supplement: ciz499_suppl_Supplementary_Table_S3 [file ciz499_suppl_supplementary_table_s3.docx]

**Supplementary Table S3:**

**Mortality in relation to various cut-off levels for ferritin and AST values.**

| **Peak ferritin** | |  |  |  |
| --- | --- | --- | --- | --- |
| **Cut-off (microg/L)** | **OR** | **N below cut-off (Alive/Dead)** | **N above or equal to cut-off (Alive/Dead)** | **P-value^a^** |
| 5 000 | 3.91 | 10 (9/1) | 66 (46/20) | 0.27 |
| 10 000 | 4.25 | 19 (17/2) | 57 (38/19) | 0.076 |
| 20 000 | 3.09 | 32 (27/5) | 44 (28/16) | 0.068 |
| 40 000 | 7.31 | 53 (45/8) | 23 (10/13) | 0.00051 |
| 100 000 | 10.67 | 65 (52/13) | 11 (3/8) | 0.0010 |
|  |  |  |  |  |
| **Peak AST (aspartate aminotransferase)** | | |  |  |
| **Cut-off (U/L)^*^** | **OR** | **N below cut-off (Alive/Dead)** | **N above or equal to cut-off (Alive/Dead)** | **P-value^a^** |
| 200 | 17.38 | 44 (43/1) | 132 (94/38) | <0.0001 |
| 500 | 13.45 | 87 (83/4) | 89 (54/35) | <0.0001 |
| 1 000 | 10.87 | 109 (101/8) | 67 (36/31) | <0.0001 |
| 2 000 | 11.05 | 129 (116/13) | 47 (21/26) | <0.0001 |
| 5 000 | 31.59 | 153 (133/20) | 23 (4/19) | <0.0001 |
| ^a^P-value from Fisher’s Exact Test.  All with peak aspartate aminotransferase >10,000 U/L died, giving an OR = infinity. | | | | |
